# Understanding the Effect of Listening to Music, Playing Music, and Singing on Brain Function: A Scoping Review of fNIRS Studies

**DOI:** 10.3390/brainsci14080751

**Published:** 2024-07-26

**Authors:** Keya Ding, Jingwen Li, Xuemei Li, Hui Li

**Affiliations:** 1 Shanghai Institute of Early Childhood Education, Shanghai Normal University, Shanghai 200233, China; keya@shnu.edu.cn (K.D.); 1000527447@smail.shnu.edu.cn (J.L.); xuemeil@shnu.edu.cn (X.L.); 2 Lab for Educational Big Data and Policymaking, Ministry of Education, Shanghai 200234, China; 3 Key Laboratory of Child Development and Learning Science, Ministry of Education, Research Center for Learning Science, Southeast University, Nanjing 210096, China; 4 Faculty of Education and Human Development, The Education University of Hong Kong, Hong Kong, China

**Keywords:** music, listen, sing, play, fNIRS, brain function

## Abstract

Music is integrated into daily life when listening to it, playing it, and singing, uniquely modulating brain activity. Functional near-infrared spectroscopy (fNIRS), celebrated for its ecological validity, has been used to elucidate this music–brain interaction. This scoping review synthesizes 22 empirical studies using fNIRS to explore the intricate relationship between music and brain function. This synthesis of existing evidence reveals that diverse musical activities, such as listening to music, singing, and playing instruments, evoke unique brain responses influenced by individual traits and musical attributes. A further analysis identifies five key themes, including the effect of passive and active music experiences on relevant human brain areas, lateralization in music perception, individual variations in neural responses, neural synchronization in musical performance, and new insights fNIRS has revealed in these lines of research. While this review highlights the limited focus on specific brain regions and the lack of comparative analyses between musicians and non-musicians, it emphasizes the need for future research to investigate the complex interplay between music and the human brain.

## 1. Introduction

Music, as described by Plato, holds an unparalleled power in reaching the depths of the human soul and bestowing grace upon it [1]. The influence of music on the brain is pervasive, with its melody and rhythm impacting cognitive functioning during interactions [2]. This influence has been harnessed in various therapeutic settings, from enhancing attention and working memory through music listening to treating depression with music interventions [3,4,5]. The rise of music therapy in psychiatric and neurodevelopmental disorders has spurred investigations into the neural mechanisms underlying musical interactions [6]. While previous neuroimaging studies have primarily focused on listening to music and singing, playing music has not received as much attention. This gap is significant because playing music can also profoundly affect brain function. In the past decade, many studies have utilized functional near-infrared spectroscopy (fNIRS), a cutting-edge neuroimaging tool with a high ecological validity, to examine brain activities while listening to music, singing, and playing music, especially in younger populations. However, no systematic review has been conducted to synthesize these fNIRS studies on the varying effects of music engagement (listening to it, singing, and playing it) on brain functions. This scoping review aims to fill this research gap by synthesizing the research on brain function while listening to music, singing, and playing music published from January 2013 to March 2024. By exploring these studies, we seek to enhance our understanding of how different musical activities distinctively shape our brains.

### 1.1. Music Engagement Shapes Human Brain

The significant effect of music on the human brain is well documented in the literature. Music, as a form of art closely tied to the human psyche, serves as a vital intelligence that conveys emotions and information while also instigating changes within individuals. Notably, studies have shown that the structure and function of the brains of music creators, including composers, performers, and singers, undergo transformations as they engage with music. These individuals have served as models for studying brain plasticity, with distinct differences observed in brain structures among professional, semi-professional, and non-professional musicians, particularly in areas related to motor skills, auditory processing, and the cerebellum [7]. For instance, research has demonstrated that children as young as 6 engaging in 15 months of instrument training can induce anatomical changes in brain regions associated with music [8]. Furthermore, differences in brain structure and function have been observed among musicians playing different instruments, such as pianists and violinists, showcasing varied effects on brain areas involved in movement and coordination [9].

In addition to music creators, the brain function of music listeners also undergoes changes during the process of listening to music. The ability to perceive and interpret music is enhanced through this process, primarily involving regions in the auditory cortex and frontal lobes within the temporal lobe [10,11,12]. Studies have indicated that the dynamics of brain activity in motor and frontal cortical areas can be observed when individuals listen to music, showcasing the intricate engagement of music with the brain, impacting functions and structures like the auditory system, sensorimotor system, and motor networks, influenced by individual differences [13].

### 1.2. Different Ways of Engagement and Divergent Impacts

The ways in which people engage with music can be categorized into three main forms: listening to music, singing, and playing music. Each form of engagement requires individuals to utilize different abilities to interact with music. Listening to music simply involves the ability to listen [14], while singing and playing instruments require the coordination of multiple abilities. Singing involves a connection between auditory processing and motor control to achieve accurate pronunciation [15], while playing an instrument requires coordination between hearing, vision, and movement [16].

Different types of musical engagement have varying effects on the brain, influenced by the diverse components of music, including both basic and formal elements. For example, music with lyrics activates a wider range of brain regions compared to instrumental music, suggesting the integration of various cortical regions during emotional music processing [17]. The processing of rhythm information in the brain engages a complex network of regions separate from those responsible for pitch, melody, and timbre perception. Different styles and elements of music trigger unique brain responses, leading to distinct changes in brain activity while listening to music or performing [18]. Previous reviews have consolidated and examined research on the relationship between music and the brain. Sachs et al. (2015) extensively discussed how changes in different elements of music, such as lyrics, emotions, style, genre, rhythm, etc., can evoke diverse brain responses [19]. Another detailed review found activation in specific brain regions in response to familiar versus unfamiliar music [20]. Koelsch et al. (2003) discovered through ERPs that music-syntactic processing elicits different patterns in females and males [21]. Additionally, music can influence the function and structure of various brain regions, including the auditory system, sensorimotor system, and motor network. Long-term music training can have distinct effects on different age groups [7,13]. Individuals with extensive instrument training show more activity in their temporal lobe cortex and premotor cortex when listening to music compared to those without musical training [16]. Reviews on the therapeutic effects of music also suggest that music has lasting impacts on the brain [22,23], highlighting the intricate relationship between music and the brain and how specific musical elements can evoke diverse responses.

In summary, the literature reviewed highlights the profound impact of music on the human brain, influencing both brain structure and function. Music creators, musicians, and listeners all experience unique changes in brain activity when engaging with music, showcasing the versatility and complexity of music’s effects. By understanding how music shapes the brain, researchers can gain valuable insights into the neural mechanisms underlying musical perception and processing, providing a foundation for further exploration into the therapeutic potential of music in various cognitive and emotional contexts.

### 1.3. The Current Study

There is a growing body of research exploring the complicated relationship between music and brain activity, with a particular focus on the neurological responses elicited during music listening. Previous review studies have explored the effects of emotions in music and familiarity with music on brain function [19,20,21], the therapeutic effects of listening to music [22,23], cognitive performance after listening to music [24], physiological reactions while listening to music [25], the effect of singing on health [26], and the neural mechanisms of singing [27]. Additionally, investigations into musical training have shed light on its influence on brain function and structure [7,13,16]. While these reviews have individually explored the effects of listening to music, singing, and playing instruments (LSP) on humans, they have not provided a comprehensive discussion encompassing all aspects of musical engagement. Moreover, the studies included in these reviews primarily relied on behavioral measurement methods and established neuroscience techniques like EEG, fMRI, and PET, neglecting the potential insights that could be gained from functional near-infrared spectroscopy (fNIRS). This technology offers a more ecologically valid approach, extending research opportunities to younger age groups and presenting a more systematic exploration of the connection between music and the brain. Therefore, the present study aims to fill this research gap by reviewing the studies on the effects of LSP on the brain using fNIRS from 2013 to 2024. In particular, the following question guided this scoping review:

*How do listening to music, singing, and playing musical instruments impact brain function, as indicated by studies utilizing fNIRS?*

## 2. Methods

This study aims to integrate the effects of music on brain function based on fNIRS neuroimaging techniques through a scoping review. Through scoping review of the existing relevant research, we can understand the current research topic, main research objects, research methods, and results, as well as highlight the brain function of individuals exposed to music in different ways (including listening to it, singing, and playing it). According to the scoping review framework proposed by Arksey and O’Malley (2005), this study adopts the following five steps for research: (1) definition of research question; (2) identification of relevant studies; (3) study selection; (4) data charting; and (5) data collation, summarization, and reporting of results [28].

### 2.1. Step 1: Research Question Definition

The main question of present review are as follows:

(1) What are the effects of LSP on human brain function?
(2) How do differences in musical features while LSP affect brain function?
(3) What role do individual differences play in the effect of LSP on brain function?
(4) What new insights has fNIRS brought to the study of the relationship between LSP and human brain function?

### 2.2. Step 2: Identification of Relevant Studies 

This review was conducted using three databases, including Web of Science (https://access.clarivate.com/wos/, accessed on 30 March 2024), Science Direct (https://www.sciencedirect.com, accessed on 31 March 2024), and PubMed (https://pubmed.ncbi.nlm.nih.gov, accessed on 31 March 2024), in March 2024. The goal of the literature search was to find all the studies on the effects of music on brain function based on fNIRS neuroimaging techniques published in past decade between 2013 and 2024. The present study defines the activities through which music affects the brain as “listening” to music, “singing”, and “playing” music. Therefore, two Boolean operators (AND and OR) and two keywords (music (listen OR sing OR sang OR play OR perform) and fNIRS) were combined to extract relevant information.

### 2.3. Step 3: Study Selection

The present study established dedicated inclusion criteria to select appropriate articles for the review (Table 1). The following were the inclusion criteria: (1) articles that were available as full texts and published during 2013–2024; (2) articles that were peer-reviewed; (3) articles written in English; (4) studies in which the participants did not have neurological disorders; (5) studies whose results included the effect of music on brain function; (6) studies including one or two of the three forms; and (7) studies using fNIRS or multimodal techniques including fNIRS.

The exclusion criteria were as follows: (1) articles without the original text; (2) articles without results; (3) articles that were not empirical research; and (4) articles that involved any ability beyond the three aforementioned abilities: listening to music, singing, and playing music.

**Figure 1:**
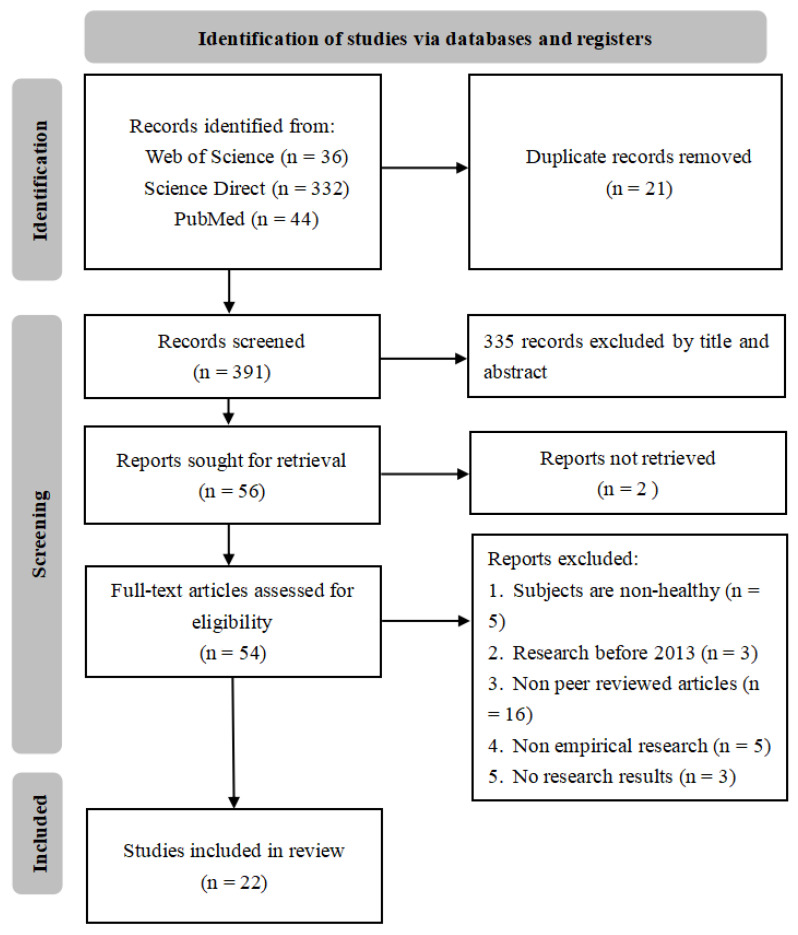
PRISMA flow diagram of literature search and data charting.

Based on inclusion and exclusion criteria, we first searched the literature by entering keywords in the aforementioned literature database, resulting in 412 articles. Not counting the 21 repeat articles, there were 391 articles. Next, we screened these articles based on the inclusion criteria: 335 articles were excluded based on their titles and abstracts; 2 articles were excluded as we were unable to retrieve the original text; 5 articles were excluded as the participants did not meet the requirements; 3 articles were excluded as they were published before 2013; 16 articles were excluded as they had not undergone peer review; 5 articles were not empirical research; and 3 articles did not have research results. The above articles were excluded as they did not meet the inclusion criteria. Finally, 22 studies on the effects of music on brain function based on fNIRS neuroimaging techniques qualified for our review. The full process of selecting the relevant studies is detailed in the PRISMA flow diagram in Figure 1.

### 2.4. Step 4: Data Charting

This present scoping review charts the data from the 22 articles that were ultimately included. The following information from these articles was extracted: authors, year of publication, country, participants’ information (sample size, gender, age, and characteristics), methods (behavior, brain area, and music materials), results, and limitations. The data graph was constructed based on three activities by which music affects the brain. There are a total of 15 articles on listening to music, 2 articles on singing, and 5 articles on playing music. Table 2 summarizes the information of these studies.

### 2.5. Step 5: Data Collation, Summarization, and Reporting of Results

Most of the selected research came from China (n = 6), South Korea (n = 4), America (n = 4), and Japan (n = 3). According to the purpose of this scoping review, the analysis focuses on: (1) characteristics of the participants; (2) the auditory materials/instruments/singing forms used in the research; and (3) the effect of music on human brain function.

## 3. Results

**Figure 2:**
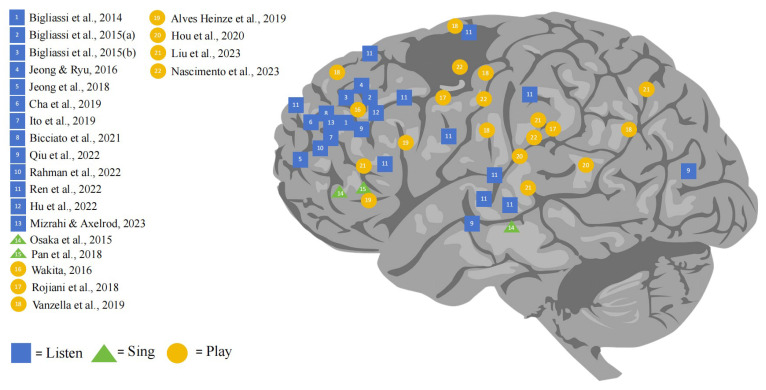
The results of the effect of fNIRS-based LSP on human brain function [29,30,31,32,33,34,35,36,37,38,39,40,41,42,43,44,45,46,47,48,49,50].

The present study reviewed 22 journal articles on the effect of music on brain function based on fNIRS neuroimaging techniques from 2013 to 2024. The results of this review are classified according to these and elaborated separately. A summary of the most important characteristics is provided in Figure 2. This section reports the effects of different musical activities on human brain function to address the three research questions.

### 3.1. The Effect of Listening to Music on Human Brain Function

A total of 15 studies on listening to music were included. These studies concerned the variables of musical features and individual differences and explored their role in the effect of music on the brain.

#### 3.1.1. The Effect of Musical Features on Brain Function

One of the studies examined the role of music rhythm in interpersonal coordination. The rhythm of music can coordinate interactions between people. For example, the intensity of the MFC-IBS under rhythmic conditions was significantly higher than that under non-rhythmic conditions, and this phenomenon was more pronounced under the high loudness [42].

Four studies explored the discrepancies in the effect of diverse music genres on the brain. Folk music significantly activated the male PFC [29]; motivational songs could trigger larger PFC activities than calm songs [31]; and classical music could significantly increase the activation level of the dlPFC compared to the mPFC, and this phenomenon became more pronounced when continuously listening to music [32]. Although this phenomenon could also be observed when listening to electronic music, the activation of the dlPFC was significantly lower than when listening to classical music [32]. Comparing classical music, pop music, and instrumental music, it was found that when listening to classical music and instrumental music, the response of the right PFC in the brain is stronger, and the same activity pattern occurred upon listening for a second time [40]. This study also found that listening to sad music triggers higher-intensity activities in the PFC compared to cheerful music.

Additionally, the complexity and logic of musical materials can affect brain activation levels. Firstly, a study investigated the brain activity of individuals as they determined melody contours while listening to music [35]. The findings revealed that as the complexity of the music increased, the activation level of the BA10 brain area also gradually increased. This indicates that this complex judgment task poses greater difficulties for individuals. Secondly, the judgment of musical logic depends on a person’s cognitive level. However, research has found no significant difference in prefrontal cortex (PFC) activity between listening to normal classical music and scrambled classical music [43].

In addition, dissimilarities in auditory materials (music and noise) can affect the degree to which the brain is biased when processing information. Research has found that the right hemisphere lateralization accounts for about 75% when listening to music, and the deviation rate when listening to noise was about 65%. If the noise level was slightly lower than the music, the subjects exhibited complete right hemisphere laterality [30].

#### 3.1.2. The Effect of Individual Differences on Brain Function

Individuals with physiological and cognitive dissimilarities exhibit different brain activities when listening to music. Gender and age are the main research topics in terms of physiological differences. Comparing the activity of the PFC among individuals of different genders when listening to different types of music, it was found that when men heard their favorite or inspirational songs, the blood flow of the PFC significantly increased, but this performance was not observed in women [29]. Similarly, comparing the brain function of males and females, it was found that listening to classical music can activate the dlPFC, and the activation level of males is higher than that of females [32]. The effect of gender was not only reflected in the process of listening to music but also after listening to music [31]. When men listen to motivational and calming songs, the oxygen content of the dlPFC was significantly higher than that of the mPFC; after listening to music, the dlPFC activity level of males was significantly higher than that of females. This indicates that music could activate the PFC and produce stronger effects in males [31].

The discrepancies in brain function caused by age mainly have two aspects. The first of which is the difference in cognitive level caused by age. In the task of melody contour recognition, the activity intensity of the dlPFC on the right side of young people was higher than that of elderly people [33]. Secondly, the level of brain development varies among people of different age groups, and the degree of environmental influence also varies. For example, a study focused on exploring the brain activity of premature infants listening to music. When the infants listened to Mozart’s K. 448, several sets of brain functional connectivity regions were highlighted: between the right temporal lobe cortex and frontal lobe cortex, between the right temporal lobe cortex and the contralateral inferior frontal gyrus, bilateral temporal lobe regions, and between the primary somatosensory cortex and the prefrontal and left inferior frontal gyrus. The perception of music by infants involved extensive functional connections between the two hemispheres at the brain network level. When the premature infants listened to music, multiple brain networks related to the perception of music features worked together [41].

Cognitive differences include music perception level as well as music learning experience. One’s music perception level includes their familiarity with a piece of music (the music’s popularity), their musical preferences, and their expectations of music. Familiar music can make the mPFC display a greater blood flow; unfamiliar songs can cause the mPFC to exhibit hypoxia [36]. However, a study found the opposite results: the LFO of the PFC showed significant changes when subjects were listening to an unfamiliar song, especially in the left PFC, and this change significantly diminished or even disappeared upon hearing the music a second time [38]. Qiu et al. (2022) compared the effects of personally preferred music and neutral music (New Age) on the entire brain and found that the music an individual likes can activate the PFC (especially the right PFC), the right temporal lobe, and the occipital lobe [39]. An experiment with a small sample size conducted by Ito et al. (2019) found that when individuals want to continue listening to music, the PFC exhibited significant activity, but there were individual differences in activation levels [37]. Music learning experience could also affect an individual’s ability to recognize music. Audiences with long-term experience learning the piano showed significant activation of the left PFC when they experienced mismatched video and audio, but those with less training (or no experience learning the piano) did not exhibit this phenomenon [34].

In general, individual differences can trigger unique brain function when listening to music, mainly including dissimilarities in individual physiological characteristics, perceptions of music, and cognitive levels. These studies mainly focus on exploring the PFC, with a few studies involving the temporal and parietal lobes.

### 3.2. The Effect of Singing on Brain Function

Only two studies related to singing were retrieved. They both focused on the synchronicity of both sides of the brain during the singing process. Osaka et al. (2015) investigated the patterns of brain interaction for different singing forms (solo or collaborative), methods (singing or humming), and levels of visibility during collaborations (face-to-face or face-to-wall) [44]. It was found that the coherence of the left IFC and right middle temporal cortex increased more under cooperative singing conditions than under solo singing conditions. The same performance was observed during cooperative humming, but it involved more brain regions, including the left parietal cortex, IFC, right middle frontal cortex, and right middle temporal cortex. In addition, under face-to-face (FtF) conditions, the consistency of the left IFC was greater than that under face-to-wall (FtW) conditions, but the increase in consistency of the right IFC was not significant. In addition to collaborative singing, teaching singing was another form of interaction. Pan et al. (2018) compared the brain function in two methods of learning to sing and found that the IFC exhibited synchronicity between learners and teachers in interactive learning tasks [45]. This change in the IBS could predict the learning situation of learners. Obviously, the IFC is an important brain area in collaborative singing activities.

### 3.3. The Effect of Playing Music on Brain Function

A total of five studies explored the brain function while playing instruments, of which four were hyperscanning studies. There are two types of character relationships when playing musical instruments: performer–listener (P-L) and performer–performer (P-P). Performers can convey emotions to listeners by playing music. Among instruments, drumming is an ancient form of nonverbal communication. A study involving 36 participants (18 pairs) found that the right TPJ plays an important role in the entire communication process of drumming [46]. The brain function during communication via drumming was similar to that during communication via language and could even trigger stronger activity. In particular, there was a high correlation between the activation of the TPJ on the right side of the listeners and the drumbeat; the drummer had one cluster on each side of the brain, with clusters BA6 and BA1, 2, and 3 in the right hemisphere and BA6 in the left hemisphere. FtF performance can convey emotions, and using electronic media to convey information can also achieve this effect. Hou et al. (2020) played 12 violin performance videos to 16 female participants and found that both the violinist and the listeners had IBC activation (the left TPJ, right IFC, and central posterior cortex) during the task, and this correlation was significantly stronger during the later observation period (>50 s) than during the early observation period (0–50 s) [49]. And the IBS was found to be correlated with the level of popularity.

In research on P-P relationships, the TPJ is the focus of attention. A team drumming study found that under team-centered conditions, the TPJ and mPFC in the left brain exhibited higher interpersonal neural synchrony [50]. No such phenomenon was observed under random and confocal conditions. A study on violin duets also found the activation of the TPJ [47]. During the duet process, there was significant activation in the sensorimotor areas and the TPJ on both sides of the brain, with the second violinist’s brain activation level higher than that of the first violinist. It can be understood that regardless of the type of relationship, the TPJ is a key brain area to explore, as it is an important area for exploring interpersonal communication problems.

The brain function during the process of learning musical instruments has also been explored. A study investigated the changes in the PFC hemodynamic signals while learning the piano [48]. Adults without experience in keyboard instruments were required to complete 10 identical keyboard practice tasks (practicing three arpeggios: IMa, VIm, and IVMa for 30 s). The study found that the activation level of the PFC significantly increased from the second block to the third block, reached its peak in the seventh block, and then stabilized and decreased. However, only one channel (OFC) showed significant activation during the task. Throughout the learning process, the OFC activation gradually decreased with increasing familiarity, presenting an inverted U-shaped change.

## 4. Discussion

This scoping review reveals a stratified landscape of neuroscientific inquiry into music’s effects on the brain, delineated by modes of musical engagement and underscored by functional near-infrared spectroscopy (fNIRS). Benefiting from fNIRS’s high ecological validity, portability, low cost, non-invasiveness, and non-ionizing properties, a further exploration of the relationship between people and music can be achieved. As a brain imaging technique suitable for capturing motion states, fNIRS can effectively collect brain activity while playing instruments and singing. Additionally, fNIRS has been used to study the differences in brain synchronization and activation during multi-person music interactions. In particular, this review uncovers four critical themes, as outlined below.

### 4.1. The Responses of Passive and Active Music Experiences in Relevant Brain Areas

The research on passive music listening predominantly zeroes in on the prefrontal cortex (PFC), a nexus of pathways influencing emotional regulation and mnemonic processes. This localization draws from the understanding that the PFC modulates internal responses to music’s emotive payloads, with ramifications for our affective and recollective circuits [51]. The PFC’s engagement with music is not monolithic; it reflects an individual’s unique psychological tapestry—whereby their memories, experiential imprints, and personality traits modulate their musical processing [52]. In contrast, active participation in music—through singing or instrumental performance—tends to pivot around the study of cerebral synchrony within dual interactions. Singing-centric studies focus on the integrative cognitive operations within the inferior frontal cortex (IFC), a subset of Broca’s area involved in mirroring and linguistic processing. These findings imply that communal singing or vocal training fosters mutual cognitive alignment, facilitating an empathetic grasp of language and actions [53,54]. For instrumentalists, the emphasis shifts to the visual reliance in beginners and the temporoparietal junction’s (TPJ) role in coordinated action and empathic understanding during interactive performances [55,56].

### 4.2. The Lateralized Brain in Music Perception and Processing

This review casts light on the distinct influences that musical components—whether basic or formal elements—exert on brain function. The auditory cortex’s bilateral response illuminates the interplay of attention and emotional valuation in music perception. Specifically, the right hemisphere’s preeminence in processing melodic contours vies with the left hemisphere’s competence in temporal resolution and speech-related processing [57,58]. These hemispheric specializations evoke lateralization in auditory perception, illuminating the cerebral mechanics of individual experiences of music.

### 4.3. Individual Variations in Neural Responses to Music

This review underscores the variability in listening to music and playing instruments, which is contingent upon individual differences. The evidence suggests the listener’s cognitive schema and experiential background subtly tint their neural response during musical activities—a notion that interfaces with research into cognitive flexibility, inhibitory control, and the reception of pitch patterns [34,35]. Particularly noteworthy is the personalized neural footprint evident in PFC activation patterns, in which familiarity and preference for musical stimuli emerge as significant modulators. This observation aligns with findings demonstrating differential mPFC engagement depending on the novelty of musical stimuli—a testament to the brain’s dynamic role as an interpreter of musical semantics [20,59]. Lastly, when discussing the interplay among individual traits, music perception, and brain activity, the discussion extends beyond subjective cognitive nuances to incorporate broader physiological and demographic variables. Here, attention is given to how age and gender may sculpt neurodevelopmental trajectories and the processing of musical stimuli, respectively, hinting at the need for targeted investigations into such differences [60,61].

### 4.4. Neural Synchronization in Musical Performance

This scoping review delves into the effects of singing and corroborates the IFC’s pivotal role in observational and predictive cognitive functions. The nature of visual stimuli and the degree of cooperative engagement during singing—the interactional depth in choir performances or teaching scenarios—propagates distinct neural correlates, suggesting a relationship between the cognitive integration of musical activities and neural synchronization within the IFC [44,45]. In the realm of instrumental performance, the temporal progression and social constructs of interactions when playing music draw forth nuanced cerebral responses, particularly in the TPJ and sensorimotor cortices. Such neural activity not only supports the mechanics of skilled movement but also indicates social and emotional engagement during ensemble play. These findings prompt intriguing questions about the brain’s temporal dynamics and the influence of social context on musical performance. In playing contexts, relational dynamics, whether between performers or between performers and the audience, are clarified through differentiated cerebral activation patterns, illustrating the two-way street of musical communication. For performers, synchrony in the TPJ may predict an audience’s favorable reception of a piece of music, potentially reflecting the transfer of emotional and conceptual intentions beneath the musical veneer [47].

## 5. Conclusions, Limitations, and Future Research

### 5.1. Conclusions

In synthesizing the current literature on the neural effects of music as captured by functional near-infrared spectroscopy (fNIRS), this scoping review has delineated the distinct neural mechanisms engaged during passive listening and active musical participation. The review has revealed that listening to music predominantly activates the prefrontal cortex (PFC), highlighting its relevance in auditory processing. In contrast, active musical expression, such as singing and playing instruments, is associated with synchronization processes within the brain, engaging the inferior frontal cortex (IFC) and the temporoparietal junction (TPJ).

Moreover, the review has elucidated how various musical features—including emotional content, rhythmic intensity, and complexity of auditory inputs—affect the brain’s lateralization of sensory processing (LSP). Crucially, individual demographic and cognitive differences have been found to modulate responses to musical stimuli, demonstrating the personalized nature of music perception and performance. Such differences manifest as divergent neural responses and subjective musical experiences, which are contingent upon factors such as age, gender, cognitive capacity, musical expertise, and personal preference.

Despite the insights provided, the review also highlights several limitations within the current body of research. There is a noted predilection for studies to focus on select brain regions, such as the PFC in listening tasks and the IFC and TPJ in tasks involving active musical engagement, potentially neglecting the broader global effects of music on the brain. Comparative studies concerning the playing of different musical instruments remain scarce, signaling a gap in our understanding of the full spectrum of music features on brain function. Additionally, while an emphasis on individual differences has predominated listening studies, future research in singing and playing music should equally consider the influence of these characteristics.

### 5.2. Limitations and Future Research

This scoping review is not without limitations. First, it is important to note that the review was constrained to studies using functional near-infrared spectroscopy (fNIRS), which may not capture all the intricate neural mechanisms associated with music perception and cognition. Future studies should expand the scope to include other neuroimaging modalities such as fMRI, EEG, MEG, and PET. This can provide a more comprehensive understanding of the neural mechanisms involved in music perception and cognition, capturing the intricate processes that fNIRS alone may not detect. Second, the literature search was limited to the period from 2013 to 2024. This temporal limitation may have excluded relevant studies published outside of this range, potentially impacting the comprehensiveness of the review. Future research should conduct an updated and continuous literature review that includes studies published before 2013 and after 2024. Third, there was a language requirement for the studies to be written in English, which raises the possibility of missing significant contributions documented in other languages and may introduce a bias in the results of the review. Future studies should include research published in multiple languages to mitigate language bias. Lastly, a common feature of scoping reviews, including this one, is that they typically do not assess the methodological quality of the studies included. This absence of quality appraisal means that the reliability of the findings could be influenced by studies of lower quality. Future reviews should include a rigorous quality appraisal of included studies to ensure the reliability and validity of findings by identifying and weighing the influence of lower-quality research. Moreover, future studies should consider diverse participant demographics to understand how music perception and cognition may vary across different populations. This can involve factors such as age, gender, cultural background, and musical training, providing a more holistic view of the neural mechanisms involved. Additionally, longitudinal neuroscience research should be conducted to track changes in music perception and cognition over time, exploring how neural mechanisms evolve with prolonged musical exposure or training. Utilizing emerging techniques such as machine learning and artificial intelligence can also enhance the analysis of complex neural data, uncovering subtle patterns and relationships.

## 6. Implications for Future Studies

The findings of this scoping review offer several compelling insights for future research in the field of preschool music education and the relationship between young children and music.

Firstly, this review underscores the pivotal role that understanding brain functionality plays in music education research. Despite the breadth of the literature reviewed, only one study focused on infants, highlighting a significant gap. To address this, future research should prioritize exploring the functional connections between different brain regions during music processing. It is crucial to study how these brain connectivity patterns evolve with age and how environmental influences affect these changes. Longitudinal and horizontal studies would be particularly beneficial, examining the development of functional connectivity from infancy to adulthood and identifying individual differences.

Secondly, there is a need to diversify the music materials used in research by classifying them into more detailed categories, which could yield more nuanced insights. Additionally, factors such as gender, temperament, age, and personality should be meticulously considered, as they can significantly affect brain function during music-related activities. Differentiating subjects based on these characteristics and integrating them into future studies could provide deeper, more individualized results.

Moreover, future research should investigate the hormonal influences of testosterone and estrogen on the central nervous system, specifically regarding gender-related behavioral patterns. Expanding the demographics of studies to include a broader age range will also enhance our comprehension of the developmental trajectories of musical perception.

Ultimately, this scoping review highlights the intricate relationship between music and brain function, advocating for multidimensional research paradigms to delve into these complexities. By considering both universal and individualized aspects of music perception and production, researchers can enrich their understanding of how music influences cognitive functions, emotional well-being, and social connections. This nuanced approach promises a more profound appreciation of the sophisticated neural symphony underpinning our engagement with music as a uniquely human experience.

## Figures and Tables

**Table 1:** Inclusion/exclusion criteria.

Criterion	Inclusion	Exclusion
Scope of research	Empirical studies	Non-empirical studies (reviews, theoretical studies, or editorials)
		Secondary data analysis
Type of documents	Peer-reviewed scholarly journal articles	Non-peer-reviewed scholarly journal articles
Language	English	Other languages than English
Participant	Healthy population	Unhealthy population
Method	fNIRS	Techniques other than fNIRS
	Multimodal technologies including fNIRS	

**Table 2:** Basic information of the 22 studies.

Contact Method	Author (year)/Country	Participants		Methods		Results
		Sample Size and Age	Inclusion Criteria	Measurement of Brain Areas	Music Experiment Materials	
Listen	Bigliassi et al. (2014)/America [29]	N = 18 (male = 10) Mage = 22.25 ± 2.34 yr.	Right-handed non-musicians	PFC	Rock: *Iron Man* Folk: *Mr. Tambourine Man* Trance: *Acperience* Classical: *Air on G String* Soft Rock: *Tears in Heaven* Subjects choose: 1 preferred song and 1 motivational song	PR, MO, and FO showed gender differences on PFC.
	Santosa et al. (2014)/Korea [30]	N = 14 (male = 7) Mage = 28 ± 5 yr.	12 right-handed; 2 left-handed	Auditory cortex	*Für Elise* (by Ludwig van Beethoven)	1. Music processing: 75% R-HL; Noise processing: 65% R-HL. 2. Mixing noise in music: increased R-HL. 3. Noise level slightly lower than that of music: completely R-HL.
	Bigliassi et al. (2015a)/America [31]	N = 30 (male = 15) Male: Mage = 24.8 ± 2.4 yr. Female: Mage = 25.2 ± 3.1 yr.	Right-handed non-musicians	PFC	Two different songs in a random order: 1. A motivational song which they self-selected. 2. A calm song (Enya, *May It Be*; New Age style, 110 bpm).	Men exhibit stronger dlPFC activity while listening to music and residual sounds; Women have a higher level of excitement when listening to motivational music.
	Bigliassi et al. (2015b)/America [32]	N = 30 (male = 15) Male: Mage = 24.8 ± 2.4 yr. Female: Mage = 25.2 ± 3.1 yr.	Right-handed non-musicians	PFC	Classical music: L. Van Beethoven, *Symphony no. 6 (Pastorale), Opera 68*, 1806–1807 Techno Magnetiko	The activity of PFC is significantly influenced by exposure time, music type, and hemisphere. Classical music concerts generate more dlPFC activities (male>female).
	Jeong and Ryu (2016)/Korea [33]	N = 27 (college students = 13, male = 10, Mage = 23.54 yr.; olders = 14, male = 7, Mage = 56.07 yr.)	Right-handed non-musicians	PFC	Six contour stimuli: combination of three contours (rising, falling, and remaining unchanged), played by one of the three synthetic instruments (piano, flute, or strings).	1. The ACC of the elderly group in CIT2 was significantly lower than that under environmental noise conditions. 2. The activity of RdlPFC in young group was significantly higher than that in elderly group in CIT2.
	Wakita (2016)/Japan [34]	With extensive training: N = 9 (male = 0), Mage = 29.2 yr. With little training: N = 9 (male = 2), Mage = 28.6 yr.	Right-handed; no visual or auditory disability	Left PFC	*Mary Had a Little Lamb* *London Bridge Is Falling Down*	Under uncoordinated conditions, participants with extensive training showed significantly higher activation in the left prefrontal region.
	Jeong et al. (2018)/Korea [35]	N = 16 (male = 10) Mage = 23.5 ± 1.7 yr.	Right-handed non-musicians	PFC	Six types of contour stimuli are paired with a combination of three contours (rising, falling, and remaining unchanged) and combined into 5 cognitive tasks (CITs).	The activity of BA10 increases with the difficulty of the task; The cognitive load suddenly increases between CIT4 and CIT5.
	Cha et al. (2019)/Korea [36]	N = 56 (male = 33) Mage = 24 yr.	N/A	mPFC	5 popular songs: 2 songs higher than OSL (optimal sensory load), 2 songs lower than OSL, and 1 song at OSL.	Songs familiar to participants and songs higher than OSL can trigger more significant right side mPFC activity
	Ito et al. (2019)/Japan [37]	N = 6 Mage = 22.5 yr.	N/A	PFC	N/A	There are three different types of PFC activities that represent them wanting to listen to music.
	Bicciato et al. (2021)/Switzerland [38]	N = 6 (male = 4) Mage = 41.2 ± 12.6 yr.	Right-handed	PFC	Choose the music style that the subjects like	When listening to music for the first time, the LFO increases, which is positively correlated with PFC activation, especially in the left hemisphere. This performance disappeared the second time they listened to music.
	Qiu et al. (2022)/China [39]	N = 9 (male = 5) Mage = 31.25 yr.	Right-handed non-musicians	EEG: Occipital lobe fNIRS: frontal cortex temporal cortex parietal cortex	Neutral music, peferred music	Listening to their favorite and neutral music mainly activated their PFC (especially the right frontal lobe) and occipital lobe; In the same brain region, low-frequency bands (such as δ and θ) had more activation than high-frequency bands (such as β and γ).
	Rahman et al. (2022)/Australia [40]	N = 27 (male = 10) Mage = 19.4 ±1.5 yr.	N/A	PFC	Three types of music with 4 pieces each.	Classical music and instrumental music cause significant changes in the right and middle PFC; Sad music triggers stronger PFC activity.
	Ren et al. (2022)/China [41]	N = 10 (male = 8) Gestation age = 34.5 ± 1.0 weeks	Premature Apgar Score ≥ 7 Automated AABR	Frontal cortex temporal cortex parietal cortex	*Mozart K.448*	Premature infants’ perception of music involves a wide range of functional connections between the two hemispheres at the brain network level: the functional performance of the frontal and temporal cortical regions shows right-sided hemisphere deviation, as well as synchronization between the bilateral temporal cortical regions.
	Hu et al. (2022)/China [42]	Expt. 1: N = 40 (female) Mage = 21.82 yr. Expt. 2: N = 32 (female) Mage = 20.64 yr.	Right-handed non-musicians who have studied fewer than 3 years	Frontal cortex	Expt. 1: a metered tone sequence and a non-metered tone sequence. Expt. 2: Four kinds of meters generated by manipulating the accent and the frequency of occurrence.	The interpersonal coordination effect is better with intervals and strong tone conditions. Among them, MFC-IBS is negatively correlated with the average interpersonal delay with intervals.
	Mizrahi and Axelrod (2023)/Israel [43]	N = 24 (male = 12) Mage = 22.2 ± 2.3 yr.	2 left-handed	PFC	*Concerto No. 1* by Tchaikovsky, *Rhapsody on a Theme of Paganini* by Rachmaninov, and *Concerto No. 2* by Rachmaninov	There was no significant difference in the activity level of PFC when listening to two types of music.
Sing	Osaka et al. (2015)/Japan [44]	Singing: N = 30 (15 pairs, M-M = 8) Mage = 22 yr. Humming: N = 28 (14 pairs, M-M = 9) Mage = 21 yr.	Not familiar with each other	IFC	Three popular Japanese nursery rhymes: *Under the Spreading Chestnut Tree* *School of Killifish* *Sunset with the Evening Glow*	Singing: The increased coherence between the left IFC and the right temporal cortex during collaboration is greater than that of a single individual. Humming: The increase in coherence during the collaboration process is greater than that of a single layer, including the left parietal cortex, bilateral IFC, right frontal cortex, and right temporal cortex, with FtF>FtW.
	Pan et al. (2018)/China [45]	Student: N = 24 (male = 0) Mage = 20.58 ± 2.15 yr. Teacher: N = 1 (male = 0) Age = 24 yr.	Right-handed	IFC	*The Moon Reflection* (Lyrics: B. Peng, Music: Z. Liu and S. Yan) *A Tune of Homesickness* (Lyrics: C. Qu, Music: Q. Zheng).	In interactive learning tasks, IFC and IBS activity between learners and teachers is demonstrated, and this specific enhancement can predict learners’ behavioral performance.
Play	Rojiani et al. (2018)/America [46]	N = 36 (male = 17) M-M = 5 pairs; M-Fe = 8 pairs; F-F = 5pairs Mage = 23.8 ± 3.2 yr.	86% right-handed participants that can play drums	Frontal cortex temporal cortex parietal cortex	Drumming	The increase in drum frequency is related to the activation of TPJ in the listener’s nerves. Listening > Drumming: Right SMG, STG, BA39, BA22, and BA21. Drumming > Listening: Right BA6 and BA1,2,3; Left BA6. Drumming > Talking: Right BA40, BA22, and BA2; Left BA2 and BA40.
	Vanzella et al. (2019)/Brazil [47]	N = 10 (duets = 5, male = 5) Mage = 32.8 ± 8 yr.	Right-handed professional musicians	Regions of the motor and sensorimotor corticesTPJ	No. 37 (Prelude and Canon)	The temporal parietal and sensorimotor cortices of second violinist are significantly more activated than those of first violinist.
	Alves Heinze et al. (2019)/Switzerland [48]	N = 15 (male = 10) Mage = 21.5 ± 2.4 tr.	Right-handedparticipants without experience in keyboard or musical instruments	PFC	Three arpeggiated chords (C major, A minor and F major) Playing the piano with the left hand	The activation level of PFC in the subjects significantly increased from the second block to the third block, reached its peak in the seventh block, and then stabilized and decreased.
	Hou et al. (2020) [49]	Audience: N = 16 (male = 0) Mage = 20.313 ± 1.922 yr. Violinist: N = 1 (male) Age = 21 yr.	Right-handed non-musician	Frontal cortex temporal cortex	Performance videos of 12 works recorded by violinists	1. Violinist’s audience shows IBC in the left temporal cortex, right IFC, and central posterior cortex. IBC can show or predict popularity. 2. The correlation of the left temporal cortex is stronger when observed later (>50 s) than when observed earlier (0–50 s).
	Liu et al. (2021)/China [50]	N = 180 (male = 105) Mage = 23.14 ± 1.93 yr.	Right-handednon-musicians who werenot familiar with each other	mPFC TPJ	Played drums under 3 conditions: (1) Random drumming; (2) Team-centered conditions; (3) Focusing on metronome together	Under team-centered conditions, higher IBS was also observed in the left TPJ and mPFC; There is a positive correlation between target drum synchronization and TPJ.

Mage = mean age; M-M = male–male pairing; M-F = male–female pairing; F-F = female–female pairing; R-HL = right hemisphere lateralization; Expt. = experiment; ACC = accuracy; fNIRS = functional near-infrared spectroscopy; EEG = electroencephalogram; PFC = prefrontal cortex; mPFC = medial prefrontal cortex; IFC = inferior prefrontal cortex; TPJ = temporoparietal junction; IBS = interpersonal brain synchronization; IBC = inter-brain coherence; INS = interpersonal neural synchronization; CIT = contour identification task; LFO = low-frequency oscillator; MFC = medial frontal cortex; Italicized text: song title.
